# On four species of the genus *Argiope* Audouin, 1826 (Araneae, Araneidae) from China

**DOI:** 10.3897/zookeys.1019.59521

**Published:** 2021-02-22

**Authors:** Cheng Wang, Jiahui Gan, Xiaoqi Mi

**Affiliations:** 1 College of Agriculture and Forestry Engineering and Planning, Tongren University, Tongren, Guizhou, 554300, China Tongren University Tongren China; 2 Guizhou Provincial Key Laboratory for Biodiversity Conservation and Utilization in the Fanjing Mountain Region, Tongren University, Tongren, Guizhou, 554300, China Tongren University Tongren China

**Keywords:** DNA barcoding, orb-weaving spider, sexual dimorphism, taxonomy

## Abstract

Based on morphological and molecular evidence, *Argiope macrochoera* Thorell, 1891 from China is found to be the unknown female of *A. cameloides* Zhu & Song, 1994, the known male of *A. perforata* Schenkel, 1963 is mismatched and provisionally suggested to be the male of *A. boesenbergi* Levi, 1983, and the true male of *A. perforata* Schenkel, 1963 is described for the first time. *Argiope abramovi* Logunov & Jäger, 2015 is suggested to be a synonym of *A. perforata* Schenkel, 1963. *Argiope chloreides* Chrysanthus, 1961 and *A. vietnamensis* Ono, 2010 are newly recorded from China. The unknown male of *A. vietnamensis* Ono, 2010 is described for the first time.

## Introduction

*Argiope* Audouin, 1826 is nested in the subfamily Argiopinae Simon, 1890 together with the genera *Gea* C.L. Koch, 1843 and *Neogea* Levi, 1983, and it comprises sexually dimorphic species well known for their showy, colorful females and their unique web stabilimenta (Levi 1983; Tan 2018). *Argiope* has 89 species worldwide, and it is the most diverse genus in Southeast Asia, including New Guinea and adjacent islands (Jäger 2012; WSC 2020). The genus is rather well studied on account of a series of revisions and reviews by Levi (1983, 2004), Bjørn (1997), Yin et al. (1997), Tanikawa (2009), and Jäger (2012). However, more than one-third of its species (31) are known only from a single sex: four by males and 27 by females. Two species lack diagnostic illustrations and cannot be confidently identified. Thus, *Argiope* remains inadequately known. To date, 19 species have been recorded from China, of which three are endemic.

While examining *Argiope* specimens from southwest China, *A. chloreides* Chrysanthus, 1961, *A. vietnamensis* Ono, 2010, and the previously unknown male of the latter were recognized. An extended study of morphological and molecular evidence has revealed that *A. macrochoera* Thorell, 1891 from China is misidentified and conspecific with *A. cameloides* Zhu & Song, 1994, and that the male of *A. perforata* Schenkel, 1963 is mismatched and may be *A. boesenbergi* Levi, 1983. Moreover, the true male of *A. perforata* is revealed for the first time, and *A. abramovi* Logunov & Jäger, 2015 is synonymized with *A. perforata* Schenkel, 1963. The goals of the present paper are to revise and describe the misidentifications, mismatches, and the unknown sexes of *A. cameloides*, *A. perforata*, and *A. vietnamensis*, as well as to provide a distributional map of those species.

## Materials and methods

All specimens were collected by beating shrubs or hand collecting and were preserved in 75% ethanol, except for the specimens for DNA extraction which were preserved in absolute ethyl alcohol. All specimens were deposited in the Museum of Tongren University, China (**TRU**).

The specimens were examined with an Olympus SZ51 stereomicroscope. After dissection, the epigyna were cleared in a trypsin enzyme solution before examination and imaging. Left male palps were used for the descriptions and illustrations. Photographs of the copulatory organs and habitus were taken with a Kuy Nice CCD or an Olympus C7070 camera mounted on an Olympus BX51 compound microscope. Compound focus images were generated using Helicon Focus v. 6.7.1.

All measurements are given in millimeters. Leg measurements are given as total length (femur, patella + tibia, metatarsus, tarsus). Abbreviations used in the text and figures are as follows:

**C** conductor;

**CD** copulatory duct;

**CO** copulatory opening;

**E** embolus;

**F** flange;

**FD** fertilization duct;

**MA** median apophysis;

**PC** paracymbium;

**Pd** pendant;

**PP** posterior plate;

**S** spermatheca;

**Sc** scape;

**Sep** septum;

**Sp** spur of the median apophysis.

The total genomic DNA from spider legs was extracted using the Animal Genomic DNA Isolation Kit (Kangwei Biotech, Beijing, China) following the manufacturer’s protocols. The primer pair LCO1490/HCO2198 (Folmer et al. 1994) was used to amplify cytochrome c oxidase subunit I (COI) under the following PCR reaction protocol: initial denaturation at 95 °C for 5 min; 35 cycles of denaturation at 95 °C for 1 min, annealing at 40 °C for 1 min, and elongation at 72 °C for 30 s; and a final extension at 72 °C for 7 min. The 25 μl PCR reactions consisted of 12.5 μl of 2×Taq MasterMix or 2×Es Taq MasterMix (KangWei Biotech, Beijing, China), 1 μl of each forward and reverse 10 μM primer, 1 μl of genomic DNA, and 9.5 μl of double-distilled H_2_O. All PCR products were purified and sequenced at Tsingke Biotechnology Company (Chengdu, China).

## Taxonomy

### Family Araneidae Clerck, 1757

#### Genus *Argiope* Audouin, 1826

##### Argiope cameloides

Taxon classificationAnimaliaAraneaeAraneidae

Zhu & Song, 1994

357E358A-0E40-512E-AF12-0AB93467AAFE

**Figure 1.**
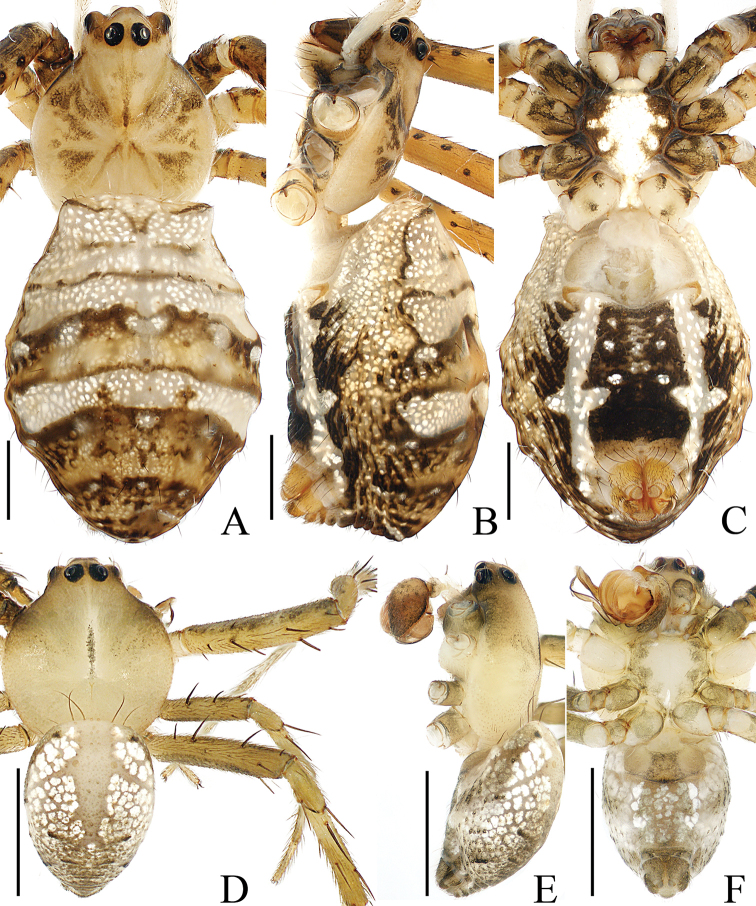
Habitus of *Argiope cameloides* Zhu & Song, 1994 **A–C** female (TRU-Araneidae-40) **D–F** male (TRU-Araneidae-37) **A, D** dorsal **B, E** lateral **C, F** ventral. Scale bars: 1.0 mm.

**Figure 2.**
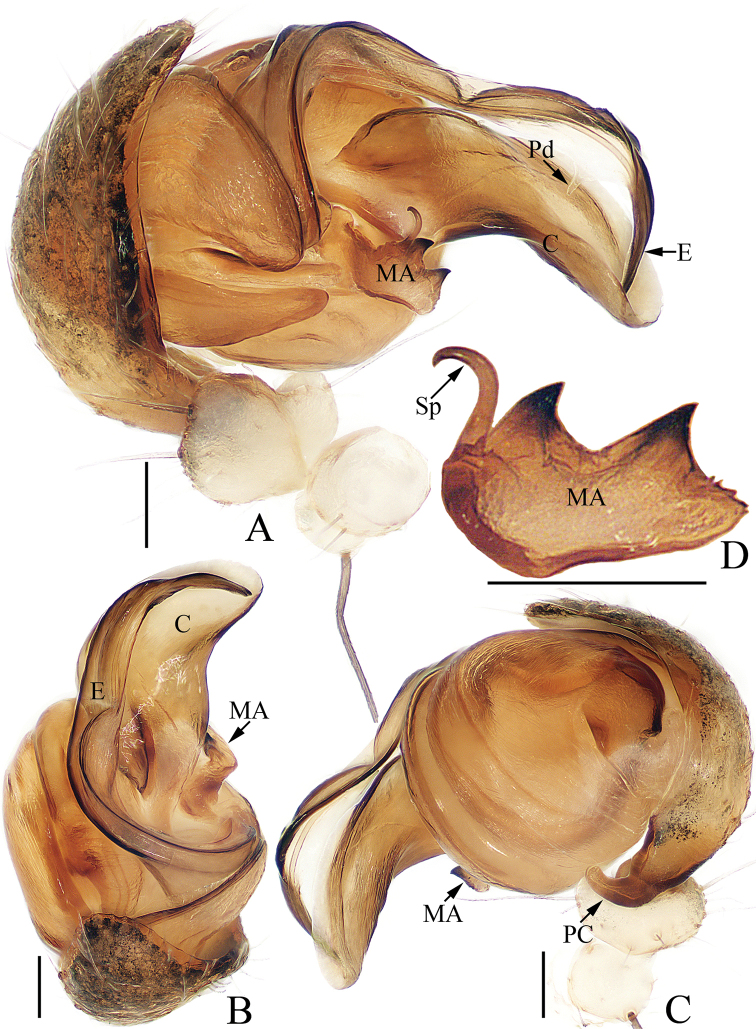
Male palp of *Argiope cameloides* Zhu & Song, 1994 (TRU-Araneidae-37) **A** prolateral **B** apical **C** retrolateral **D** median apophysis, posterior. Scale bars: 0.1 mm.

**Figure 3.**
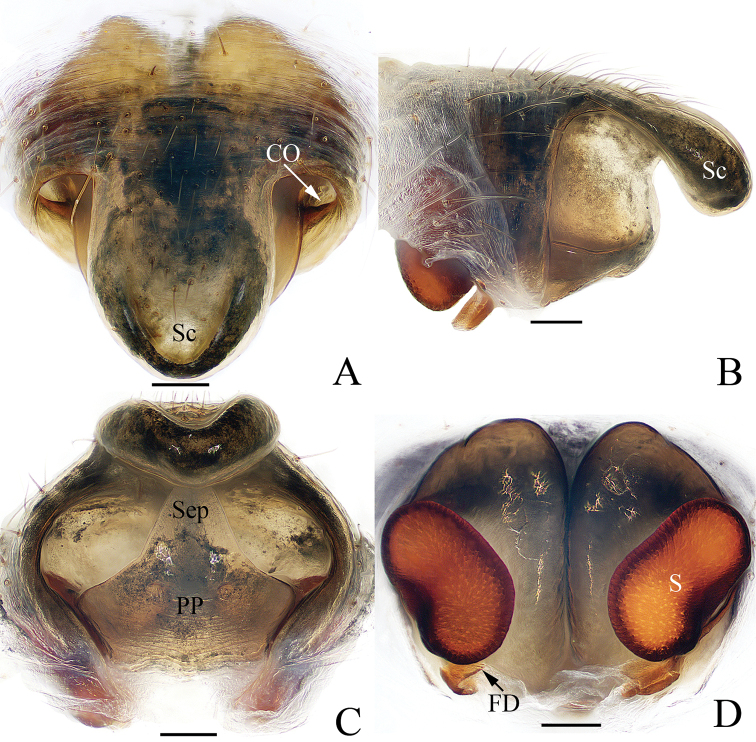
Epigyne-vulva of *Argiope cameloides* Zhu & Song, 1994 (TRU-Araneidae-40) **A** ventral **B** lateral **C** posterior **D** dorsal. Scale bars: 0.1 mm.

Figures 1 , 2 , 3 , 10

Argiope cameloides Zhu & Song in Zhu et al. 1994: 33, fig. 8A–C (♂, holotype from China, locality Jianfengling National Nature Reserve of Hainan Province and deposited in Hebei University, not examined); Yin et al. 1997: 82, fig. 14a–c (♂); Song et al. 1999: 261, figs 152P, 153J (♂); Jäger 2012: 307, figs 123–125 (♂).
A. macrochoera Yin et al., 1989: 63, fig. 3A–C (♀, the specimens from Guangdong Province of China, locality unspecified and deposited in Hunan Normal University, not examined); Yin et al. 1997: 68, fig. 2a–f (♀); Song et al. 1999: 261, figs 151Q–S, 153L (♀) (misidentified).

###### Material examined.

**China** – **Guangxi Zhuang Autonomous Region** • 4♂1♀ (TRU-Araneidae-31–35), Beihai City, Yinhai District, Yajishan Forestry Station (21°35.37'N, 109°18.41'E, ca 30 m), 12.viii.2017, Xiaoqi Mi et al. leg. • 1♀ (TRU-Araneidae-36), same locality, night of 03.x.2018, Xiaoqi Mi et al. leg. • 2♂ (TRU-Araneidae-37–38), Beihai City, Tieshangang District, Xinggang Township, Xiaomatou Village, Caobiaotang (21°33.11'N, 109°29.22'E, ca 10 m), 04.x.2018, Xiaoqi Mi et al. leg. • 2♀ (TRU-Araneidae-39–40), Fangchenggang City, Shangsi County, Wenlingshan Park, night of 06.x.2018, Xiaoqi Mi et al. leg.

###### Diagnosis.

The male of this species resembles *A. dang* Jäger & Praxaysombath, 2009 in having a similarly shaped median apophysis and broad, flat conductor, but it differs in: 1) the embolus is almost directed towards 6 o’clock apically in prolateral view (Fig. 2A), versus almost 9 o’clock in *A. dang* (Jäger and Praxaysombath 2009: fig. 38); 2) the distal end of the embolus is not expanded (Fig. 2A), versus expanded in *A. dang* (Jäger and Praxaysombath 2009: fig. 39); 3) the embolus has a distinct lamellar pendant (Fig. 2A), versus absent in *A. dang* (Jäger and Praxaysombath 2009: fig. 38). The female of the species resembles *A. macrochoera* Thorell, 1891 in having the broad epigynal scape incrassated on the posterior-lateral margin but it differs in: 1) the epigynal scape is distinctly longer than wide in ventral view (Fig. 3A), versus almost as long as wide in *A. macrochoera* (Levi 1983: fig. 19); 2) the septum is narrowest anteriorly in the posterior view (Fig. 3C), versus narrowest medially in *A. macrochoera* (Levi 1983: fig. 20).

###### Description.

**Male** (TRU-Araneidae-37). Total length 2.62. Carapace 1.46 long, 1.34 wide; abdomen 1.38 long, 1.02 wide. Eye sizes and interdistances: AME 0.10, ALE 0.06, PME 0.11, PLE 0.09, AME–AME 0.13, AME–ALE 0.05, PME–PME 0.14, PME–PLE 0.16. Legs: I 5.92 (1.68, 1.85, 1.63, 0.76), II 5.78 (1.63, 1.85, 1.54, 0.76), III 3.22 (1.02, 0.93, 0.76, 0.51), IV 4.65 (1.46, 1.39, 1.24, 0.56). Carapace (Fig. 1D, E) pale and flat, acutely narrowed anteriorly and followed by rounded thorax area, with a longitudinal brown stripe medially. Fovea linear. Chelicerae (Fig. 1F) pale, mingled with green. Endites (Fig. 1F) brown laterally and white on the inner side. Labium (Fig. 1F) pale, hairy. Sternum (Fig. 1F) heart-shaped, pale medially and green-brown laterally. Legs (Fig. 1D) pale to yellow, more or less mingled with green, armed with macrosetae. Abdomen (Fig. 1D–F) oval, dorsum with a longitudinal, branched pale-brown patch, and silver spots gradually smaller from the anterior margin; venter greyish-white, with marked silver spots median-laterally. Spinnerets brown, hairy.

Palp (Fig. 2A–D): patella with a long bristle; tibia swollen; paracymbium curved medially, with a blunt tip directed towards the bulb in retrolateral view; median apophysis looks like a swan, with two wide spurs pointed apically, and a small, slender, tapered, and curved spur; conductor broad and flat, slightly curled; embolus tapered, spiraled proximally, and curved posteriorly, with a transparent short pendant directed towards the conductor.

**Female** (TRU-Araneidae-40). Total length 6.78. Carapace 2.72 long, 2.28 wide; abdomen 4.38 long, 3.22 wide. Eye sizes and interdistances: AME 0.18, ALE 0.09, PME 0.19, PLE 0.16, AME–AME 0.14, AME–ALE 0.15, PME–PME 0.24, PME–PLE 0.28. Legs: I 13.39 (3.88, 4.25, 3.88, 1.38), II 13.14 (3.75, 4.13, 3.88, 1.38), III 7.77 (2.63, 2.38, 1.75, 1.01), IV 12.39 (4.13, 3.63, 3.38, 1.25). Carapace (Fig. 1A, B) pale yellow, narrowed, and anteriorly elevated, with brown radial markings on the thorax region, bearing sparse hairs. Fovea depressed. Endites dark at base and pale apically, labium pale to white (Fig. 1C). Sternum (Fig. 1C) heart-shaped, with radial white patches and brown margins. Legs spiny. Abdomen (Fig. 1A–C) elongate-oval, with a pair of anterio-lateral humps, a white dorsum with narrow, brown bands anteriorly, and three wide, brown, posterior bands, each bearing five silver spots; venter dark brown, with a pair of cruciform, longitudinal bands laterally and three pairs of silver white spots medially. Spinnerets yellow. Epigyne (Fig. 3A–D) with a well-developed, linguiform scape incrassated on the posterior-lateral margin; median septum lamellar, widened posteriorly and fused with posterior plate; copulatory openings located on each side of the posterior plate in posterior view; copulatory ducts invisible; spermathecae reniform, separated from each other by more than their width; fertilization ducts lamellar, posterior to spermathecae.

###### Distribution.

China (Hainan, Guangdong, and Guangxi).

###### GenBank accession numbers.

TRU-Araneidae-34: MW462189, TRU-Araneidae-35: MW462190.

###### Comments.

*Argiope cameloides* is known only from the descriptions of the holotype from Hainan, China (see Zhu et al. 1994: 33, fig. 8A–C). Because the pairing has been supported by the result of DNA barcoding, and the collection site is geographically near the type locality, we identified these specimens from Guangxi, China, as belonging to *A. cameloides*. Moreover, the female of *A. macrochoera* from China differs from the holotype in some details (the differences have also been noted by Yin et al. (1997)) and is almost identical to these *A. cameloides* specimens, and, consequently, it is proposed as the female of *A. cameloides*.

##### Argiope chloreides

Taxon classificationAnimaliaAraneaeAraneidae

Chrysanthus, 1961

09633761-E57D-5427-BE0E-16E16AFA101D

Figures 4 , 10

Argiope chloreides Chrysanthus 1961: 197, figs 5–8 (♀, female types from Indonesia, locality the environs of Mindiptana and deposited in Rijksmuseum van Natuurlijke Historie, Leiden, not examined); Tan et al. 2019: 49, figs 25–41 (♂♀, removed from Argiope chloreis).
Argiope chloreis Levi 1983: 292, figs 141–143, 146–147 (♀); Jäger 2012: 296, figs 69–72 (♀; misidentified per Tan et al. 2019: 49).

###### Material examined.

**China** – **Guangxi Zhuang Autonomous Region** • 1♂ (TRU-Araneidae-41), Chongzuo City, Longzhou County, Zhubu Township, Nonggang Village (22°27.84'N, 106°56.52'E, ca 170 m), 06.vii.2019, Cheng Wang et al. leg.

**Figure 4.**
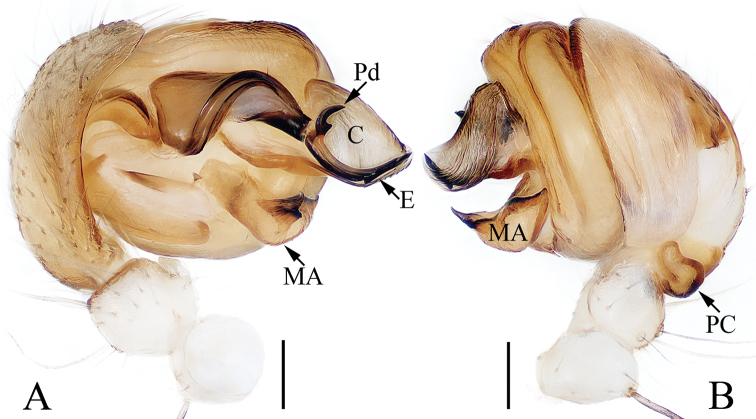
Male palp of *Argiope chloreides* Chrysanthus, 1961 (TRU-Araneidae-41) **A** prolateral **B** retrolateral. Scale bar: 0.1 mm.

###### Description.

Well described and illustrated by Tan et al. (2019).

###### Distribution.

China (Guangxi); Laos, Malaysia, Indonesia.

##### Argiope perforata

Taxon classificationAnimaliaAraneaeAraneidae

Schenkel, 1963

378DB032-38FE-5DA9-8E99-CA247746CAB4

**Figure 5.**
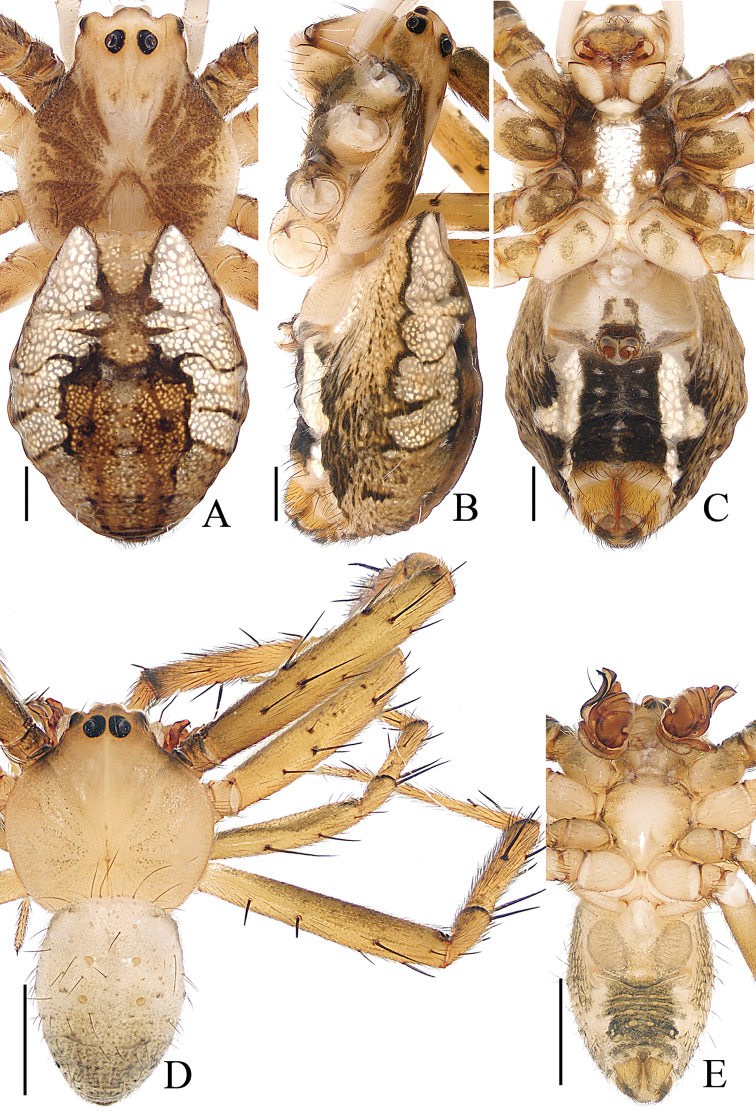
Habitus of *Argiope perforata* Schenkel, 1963 **A–C** female (TRU-Araneidae-122) **D, E** male (TRU-Araneidae-118) **A, D** dorsal **B** lateral **C, E** ventral. Scale bars: 1.0 mm.

**Figure 6.**
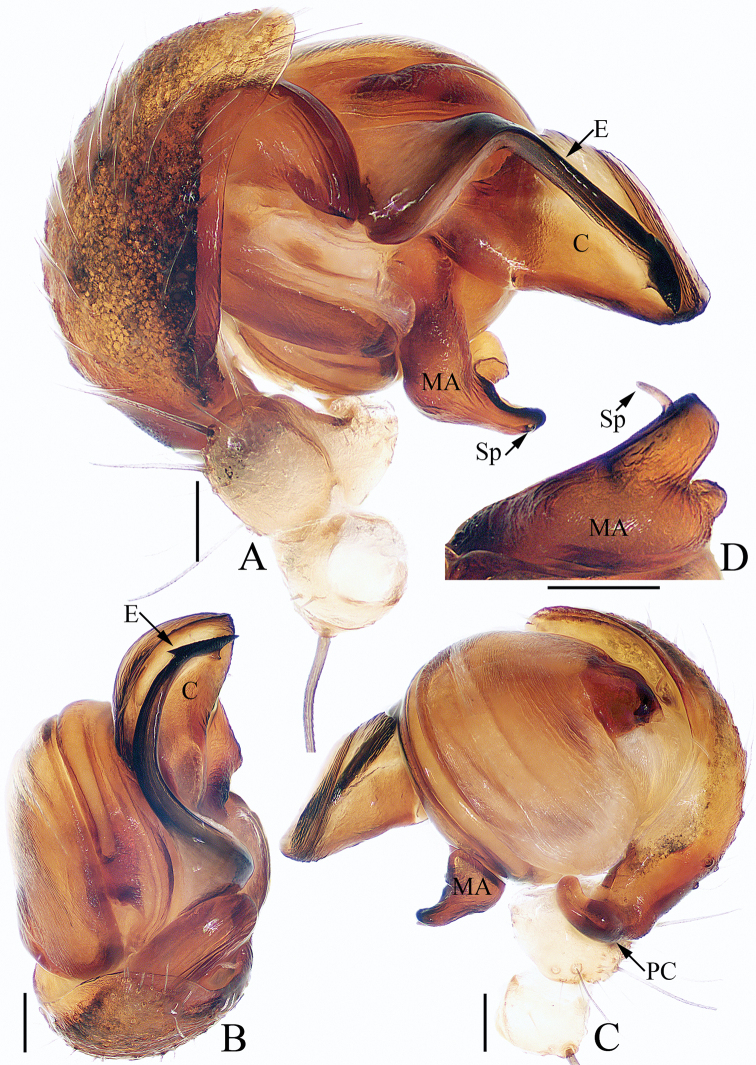
Male palp of *Argiope perforata* Schenkel, 1963 (TRU-Araneidae-118) **A** prolateral **B** apical **C** retrolateral **D** median apophysis, posterior. Scale bars: 0.1 mm.

**Figure 7.**
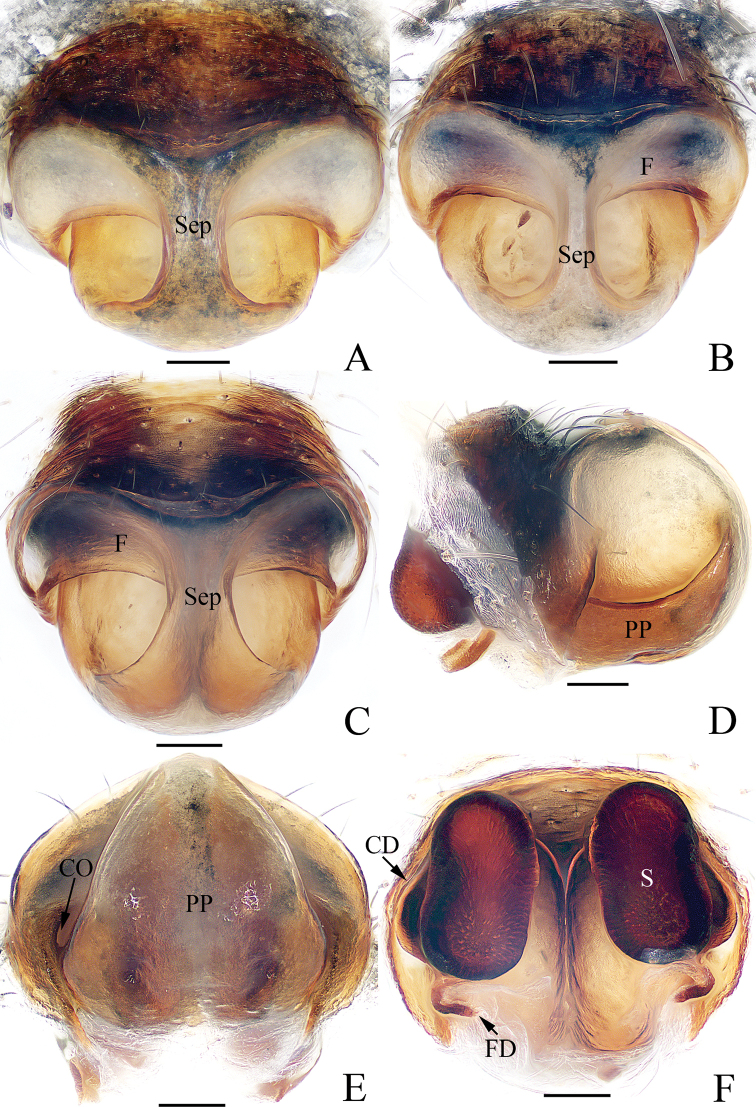
Epigyne-vulva of *Argiope perforata* Schenkel, 1963 **A**TRU-Araneidae-116 **B**TRU-Araneidae-122 **C–F**TRU-Araneidae-123 **A–C** ventral **D** lateral **E** posterior **F** dorsal. Scale bars: 0.1 mm.

Figures 5 , 6 , 7 , 10

Argiope perforata Schenkel 1963: 135, fig. 79a, b (♀, female holotype from China locality Lunan fu City, Szetchuan Province and deposited in the Museum National d'Histoire Naturelle, Paris, not examined); Levi 1983: 293, figs 162–166 (♀); Wang 1988: 101, figs 1–3 (♂, mismatched); Yin et al. 1997: 78, fig. 11a–i (♀; ♂, mismatched); Song et al. 1999: 262, figs 151V, Y, Z, 153B, M (♀; ♂, mismatched); Yin et al. 2012: 578, fig. 278a–i (♀; ♂, mismatched).
Argiope abramovi Logunov and Jäger 2015: 345, figs 1–7 (♀, female holotype from Vietnam, locality 14 km N of Kon Plong Town, Kon Plong District, Kon Tum Province and deposited in the Zoological Museum of Moscow University (ZMMU), Moscow, Russia, not examined). Syn. nov.

###### Material examined.

**China** – **Guizhou Province** • 1♂ (TRU-Araneidae-42), Tongren City, Bijiang District, Wenbi Park (27°43.26'N, 109°10.03'E, ca 460 m), 19.v.2013, Xiaoqi Mi et al. leg. • 1♂ (TRU-Araneidae-43), same locality, 10.vi.2017, Cheng Wang leg. • 3♂ (TRU-Araneidae-44–46), Tongren City, Jiangkou County, Dewang Township, Baxi Village, Nanmuping (27°51.68'N, 108°36.88'E, ca 900 m), 10.vi.2013, Xiaoqi Mi et al. leg. • 5♀4♂ (TRU-Araneidae-47–55), same locality, 14.vi.2013, Xiaoqi Mi et al. leg. • 6♀ (TRU-Araneidae-56–61), same locality, 15–16.vii.2015, Cheng Wang and Mingyong Liao leg. • 2♂ (TRU-Araneidae-62–63), Tongren City, Jiangkou County, Dewang Township, Baxi Village, Datuzu, 10.vi.2013, Xiaoqi Mi et al. leg. • 1♀ (TRU-Araneidae-64), Tongren City, Yinjiang County, Muhuang Township, Jinchang Village (28°01.37'N, 108°45.00'E, ca 1300 m), 14.vii.2013, Xiaoqi Mi et al. leg. • 1♀ (TRU-Araneidae-65), Tongren City, Songtao County, Wuluo Township, Lengjiaba Village (27°54.93'N, 108°36.70'E, ca 1150 m), 15.vii.2013, Xiaoqi Mi et al. leg. • 3♀ (TRU-Araneidae-66–68), same locality, 10.vii.2015, Cheng Wang and Mingyong Liao leg. • 3♂ (TRU-Araneidae-69–71), Qiannan Buyi and Miao Autonomous Prefecture, Libo County, Dongtang Township, Yaosuo Village, Bizuo, Maolan National Nature Reserve (25°16.37'N, 108°02.97'E, ca 550 m), 07–10.viii.2013, Xiaoqi Mi et al. leg. • 1♀ (TRU-Araneidae-72), Tongren City, Jiangkou County, near the Kaima primary school (27°50.77'N, 108°46.43'E, ca 530 m), 07.vii.2015, Cheng Wang and Mingyong Liao leg. • 2♀ (TRU-Araneidae-73–74), Tongren City, Jiangkou County, Taiping Township, Kuaichang Village, Macaohe (27°49.08'N, 108°51.52'E, ca 680 m), 08.vii.2015, Cheng Wang and Mingyong Liao leg. • 1♀ (TRU-Araneidae-75), Tongren City, Yinjiang County, Ziwei Township, Zhangjiaba Village (27°56.62'N, 108°36.60'E, ca 780 m), 12.vii.2015, Cheng Wang and Mingyong Liao leg. • 1♀ (TRU-Araneidae-76), Tongren City, Yinjiang County, Ziwei Township, Tuanlong Village (27°54.93'N, 108°42.70'E, ca 1150 m), 14.vii.2015, Cheng Wang et al. leg. • 3♀1♂ (TRU-Araneidae-77–80), Tongren City, Jiangkou County, Dengwang Township, Jinghe Village, Xujiagou (27°48.29'N, 108°37.45'E, ca 820 m), 20.vii.2015, Cheng Wang et al. leg. • 2♀ (TRU-Araneidae-81–82), Qiandongnan Miao and Dong Autonomous Prefecture, Shibing County, Yuntaishan Scenic Area (27°07.74'N, 108°06.57'E, ca 990 m), 30.vii.2015, Cheng Wang et al. leg. • 4♀1♂ (TRU-Araneidae-83–87), Qiannan Buyi and Miao Autonomous Prefecture, Libo County, Maolan National Nature Reserve (25°16.07'N, 108°59.07'E, ca 760 m), 30.iv.2016, Xiaoqi Mi et al. leg. • 1♀ (TRU-Araneidae-88), Zunyi City, Xishui County, Sanchahe Township, Sanchahe Village (28°29.04'N, 106°25.28'E, ca 800 m), 29.vii.2016, Cheng Wang et al. leg. • 4♂ (TRU-Araneidae-89–92), Zunyi City, Xishui County, Sanchahe Township, Sanchahe Village, Tiantangba (28°26.37'N, 106°24.96'E, ca 1000 m), 30.vii.2016, Cheng Wang et al. leg. • 2♀ (TRU-Araneidae-93–94), Tongren City, Bijiang District, Tianshengqiao Scenic Area (27°49.80'N, 109°12.77'E, ca 580 m), 12.v.2018, Xiaoqi Mi et al. leg. • 1♀1♂ (TRU-Araneidae-95–96), Tongren City, Shiqian County, Pingshan Township, Daping Village (27°17.97'N, 108°10.05'E, ca 720 m), 08.vi.2019, Cheng Wang et al. leg. • 1♂ (TRU-Araneidae-97), Tongren City, Songtao County, Wuluo Township, Taohuayuan Village (27°58.04'N, 108°47.73'E, ca 790 m), 29.v.2017, Xiaoqi Mi et al. leg. • 3♀ (TRU-Araneidae-98–100), Qiandongnan Miao and Dong Autonomous Prefecture, Leishan County, Fangxiang Township, Getou Village (26°24.09'N, 108°15.40'E, ca 1100 m), night of 22.vii.2017, Cheng Wang et al. leg. • 1♂ (TRU-Araneidae-101), Guiyang City, Guanshanhu District, Donglinsi Park (26°39.60'N, 106°38.00'E, ca 1300 m), 23.v.2018, Cheng Wang leg. • 1♂ (TRU-Araneidae-102), Qiandongnan Miao and Dong Autonomous Prefecture, Zhenyuan County, Dadi Township, Dadi Village (27°21.56'N, 108°12.29'E, ca 700 m), night of 23.v.2018, Xiaoqi Mi leg. • 3♀ (TRU-Araneidae-103–105), Tongren City, Shiqian County, Pingshan Township, Fodingshan Village, Yaoshang (27°20.54'N, 108°09.50'E, ca 640 m), night of 24.v.2018, Xiaoqi Mi et al. leg. • 3♀ (TRU-Araneidae-106–108), Tongren City, Yinjiang County, Yangxi Township (27°38.53'N, 108°26.43'E, ca 700 m), 17.vi.2018, Xiaoqi Mi et al. leg. • 1♂ (TRU-Araneidae-109), Tongren City, Shiqian County, Ganxi Township, near the 524 road (27°25.10'N, 108°07.97'E, ca 550 m), night of 06.vi.2019, Cheng Wang et al. leg. • 2♀ (TRU-Araneidae-110–111), Shibing County, Baiduo Township, Heichong (27°9.37'N, 108°07.40'E, ca 990 m), 20.vi.2019, Xiaoqi Mi et al. leg. • 1♀(TRU-Araneidae-112), Qiandongnan Miao and Dong Autonomous Prefecture, Shibing County, Chengguan Township (27°03.02'N, 108°7.78'E, ca 700 m), night of 21.vi.2019, Xiaoqi Mi et al. leg.; **China** – **Guangxi Zhuang Autonomous Region** • 1♀2♂ (TRU-Araneidae-113–115), Fangchenggang City, Shangsi County, Shiwandashan National Forest Park (21°53.87'N, 107°54.26'E, ca 370 m), 14.viii.2017, Xiaoqi Mi et al. leg.; **China** – **Hainan Province** – **Ledong County** – **Jianfeng Township** – **Jianfengling National Nature Reserve** • 1♀ (TRU-Araneidae-116), Mingfeng Valley (18°44.61'N, 108°51.24'E, ca 810 m), night of 12.iv.2019, Cheng Wang and Yuanfa Yang leg. • 1♀ (TRU-Araneidae-117), Sanfenqu (18°45.24'N, 108°51.57'E, ca 900 m), night of 13.iv.2019, Cheng Wang and Yuanfa Yang leg. • 1♂ (TRU-Araneidae-118), Tianchi (18°45.22'N, 108°51.53'E, ca 850 m), 14.iv.2019, Cheng Wang & Yuanfa Yang leg. • 2♀1♂ (TRU-Araneidae-119–121), Sanfenqu (18°45.24'N, 108°51.57'E, ca 900 m), night of 14.iv.2019, Cheng Wang and Yuanfa Yang leg. • 2♀ (TRU-Araneidae-122–123), Yulin Valley, Zijin Waterfull (18°44.79'N, 108°55.76'E, ca 630 m), 15.iv.2019, Cheng Wang and Yuanfa Yang leg. • 1♂ (TRU-Araneidae-124), Tianchi (18°45.22'N, 108°51.53'E, ca 850 m), night of 15.iv.2019, Cheng Wang and Yuanfa Yang leg.

###### Diagnosis.

The male of this species resembles *A. aetheroides* Yin, Wang, Zhang, Peng & Chen, 1989 in the general shape of the palp, but it differs in the distal end of embolus is slender (Fig. 6A) versus flattened in *A. aetheroides* (Yin et al. 1997: fig. 13h), and the median apophysis is bifurcated at its distal end in posterior view (Fig. 6D) versus bifurcated at the base in *A. aetheroides* (Yin et al. 1997: fig. 13i). The female of the species closely resembles *A. anasuja* Thorell, 1887 in having the epigynal flange and broad posterior plate, but it differs in the median septum being less than 1/3 of the posterior plate width in ventral view (Fig. 7A–C), versus more than 1/3 of the posterior plate width in *A. anasuja* (Levi 1983: fig. 167) and the dorsal abdomen possesses white patches laterally (Fig. 5A, B), versus has three wide, transverse white patches in *A. anasuja* (Levi 1983: fig. 170).

###### Description.

**Male** (TRU-Araneidae-118). Total length 3.65. Carapace 1.98 long, 1.90 wide; abdomen 1.90 long, 1.37 wide. Eye sizes and interdistances: AME 0.14, ALE 0.07, PME 0.15, PLE 0.12, AME–AME 0.13, AME–ALE 0.07, PME–PME 0.18, PME–PLE 0.16. Legs: I 9.59 (2.67, 3.01, 2.81, 1.10), II 9.39 (2.67, 2.95, 2.67, 1.10), III 5.05 (1.67, 1.48, 1.14, 0.76), IV 7.59 (2.48, 2.24, 2.01, 0.86). Carapace (Fig. 5D) pale pink, flat, acutely narrowed anteriorly, with indistinct, brown, radial markings on the thorax. Fovea linear, chelicerae and endites (Fig. 5E) yellow. Labium (Fig. 5E) pale. Sternum (Fig. 5E) heart-shaped, paler medially. Legs (Fig. 5D) yellow, spiny. Abdomen (Fig. 5D, E) elongate-oval, dorsum pale, darker posteriorly, with two pairs of muscle depressions medially, covered with sparse dark long hairs; venter pale laterally and green-brown medially. Spinnerets yellow, hairy.

Palp (Fig. 6A–D): patella with a long bristle; tibia swollen; paracymbium curved medially, blunt at tip; median apophysis bifurcated, with a short terminal spur; conductor flat, slightly curled; embolus enlarged at proximal end, curved almost 90° medially in prolateral view and with a lance-like tip in apical view.

**Female** (TRU-Araneidae-122). Total length 9.86. Carapace 4.67 long, 4.14 wide; abdomen 5.76 long, 4.48 wide. Eye sizes and interdistances: AME 0.26, ALE 0.15, PME 0.27, PLE 0.26, AME–AME 0.25, AME–ALE 0.34, PME–PME 0.49, PME–PLE 0.51. Legs: I 16.77 (5.13, 5.38, 4.63, 1.63), II 16.51 (5.13, 5.25, 4.50, 1.63), III 9.32 (3.25, 2.69, 2.25, 1.13), IV 14.76 (5.25, 4.38, 3.88, 1.25). Carapace (Fig. 5A, B) narrowed anteriorly and rounded in thorax region, pale yellow with brown butterfly-shaped markings, covered with sparse hairs. Fovea depressed. Chelicerae (Fig. 5C) pale yellow. Endites, labium (Fig. 5C) dark at base with pale tip. Sternum (Fig. 5C) heart-shaped, red-brown laterally, with a longitudinal white band and a pair of white spots posterio-laterally. Legs yellow to yellow-brown. Abdomen (Fig. 5A–C) suboval with a pair of anterior-lateral humps, dorsum silver with three pairs of lateral transverse brown stripes, and a broad golden marking gradually narrowing from the middle part to the terminus; venter dark-brown with a pair of white longitudinal bands laterally. Spinnerets yellow, hairy. Epigyne (Fig. 7A–F) longer than wide, with a narrow, constricted septum; the posterior plate broad, almost 3/4 of the epigynal width.

###### Distribution.

China (Hainan, Guangxi).

###### GenBank accession numbers.

TRU-Araneidae-116: MW464195, TRU-Araneidae-121: MW464196.

###### Comments.

*Argiope perforata* was originally described based on the female holotype from Sichuan, China; the illustrations are poor. It was redescribed and better illustrated by Levi (1983) who based on a specimen from Guangdong, China. Herein, the opposite sexes of the new specimens were collected from the same localities, and their pairing has been supported by the result of DNA barcoding. Additionally, the male of *A. perforata*, as described by Wang (1988), is considered to be mismatched and herein proposed to be the male of *A. boesenbergi* Levi, 1983 due to the close resemblances in palp structure. Besides, *A. abramovi* Logunov & Jäger, 2015 is almost indistinguishable from the new material of *A. perforata* in the epigynal structures and generally habitus markings, except that there are some differences in the dorsal abdomen markings; thus, *A. abramovi* is suggested as a synonym of *A. perforata*.

##### Argiope vietnamensis

Taxon classificationAnimaliaAraneaeAraneidae

Ono, 2010

211978D8-EF94-5F77-96F6-F524AB591F20

Figures 8 , 9 , 10

Argiope vietnamensis Ono 2010: 8, figs 10, 24–27 (♀, female types from Vietnam, locality TriSao, altitude 400–500 m, near Bach Ma National Park, Thua Thien Hue Province and deposited in the Arachnid Collection of the Institute of Zoology of the National Museum of Nature and Science, Tokyo, not examined).

###### Material examined.

**China** – **Guangxi Zhuang Autonomous Region** • 1♂, 1♀ (subadult) (TRU-Araneidae-125–126), Fangchengang City, Shangsi County, Shiwandashan National Forest Park (21°53.87'N, 107°54.26'E, ca 370 m), night of 14.viii.2017, Xiaoqi Mi et al. leg.; 1♀ (TRU-Araneidae-127), Chongzuo City, Jiangzhou District, Zuozhou Township, Guanghe Village (22°34.72'N, 107°24.94'E, ca 160 m), night of 03.vii.2019, Cheng Wang et al. leg.; 1♀ (TRU-Araneidae-128), Chongzuo City, Longzhou County, Kouke Village (22°18.18'N, 106°42.23'E, ca 960 m), 08.vii.2019, Cheng Wang et al. leg.; **China** – **Guizhou Province** – **Qiannan Buyi and Miao Autonomous Prefecture** – **Libo County** • 1♀ (TRU-Araneidae-129), Xiaoqikong Scenic Area (25°15.28'N, 107°42.96'E, ca 580 m), 08.vii.2017, Xiaoqi Mi et al. leg. • 5♀ (TRU-Araneidae-130–134), Dongtang Township, Yaosuo Village, Maolan National Nature Reserve (25°16.37'N, 108°02.97'E, ca 550 m), 07–10.viii.2013, Xiaoqi Mi et al. leg. • 1♂ (TRU-Araneidae-135), Weng’ang Township, Jilong Village, Maolan National Nature Reserve (25°13.53'N, 107°56.18'E, ca 840 m), 10–11.viii.2013, Xiaoqi Mi et al. leg.

###### Diagnosis.

The female was clearly diagnosed by Ono (2010). The male of this species resembles *A. minuta* Karsch, 1879 in having the elongated median apophysis terminally divided into two branches, of which the inner branch bears a short spur. It differs in having the embolus being completely visible in prolateral view (Fig. 9A), versus partly hidden by the conductor in *A. minuta* (Levi 1983: fig. 210), and in the median apophysis lacking a proximal protrusion (Fig. 9A), versus having a distinctly proximal protrusion in *A. minuta* (Levi 1983: fig. 210).

###### Description.

Female described and illustrated by Ono (2010).

**Figure 8.**
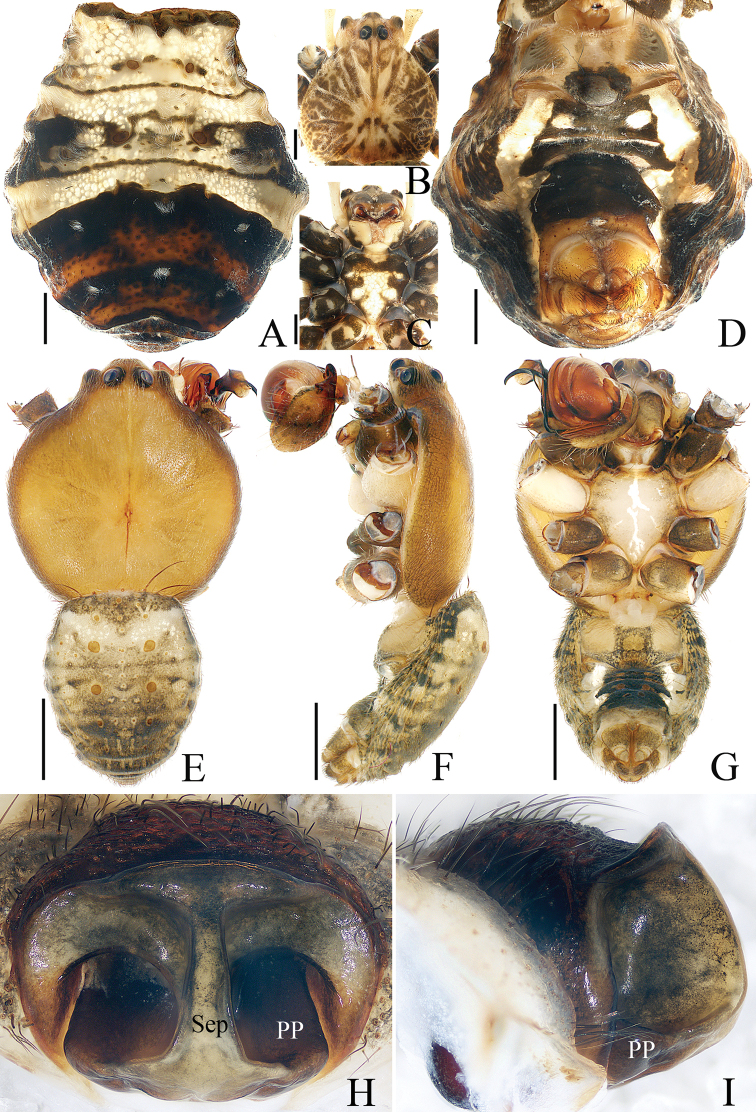
*Argiope vietnamensis* Ono, 2010 **A–D** female habitus (TRU-Araneidae-126) **E–G** male habitus (TRU-Araneidae-125) **H, I** epigyne (TRU-Araneidae-127) **A, B, E** dorsal **C, D, G, H** ventral **F, I** lateral. Scale bars: 1.0 mm (**A–G**); 0.1 mm (**H, I**).

**Male** (TRU-Araneidae-125). Total length 5.21. Carapace 3.10 long, 2.88 wide; abdomen 2.37 long, 1.93 wide. Eye sizes and interdistances: AME 0.23, ALE 0.12, PME 0.24, PLE 0.21, AME–AME 0.23, AME–ALE 0.14, PME–PME 0.24, PME–PLE 0.22. Legs were used for DNA extraction. Carapace (Fig. 8E, F) yellow-brown, narrowed anteriorly, rounded medio-posteriorly, and flattened dorso-ventrally, covered with short thin hairs. Fovea linear. Chelicerae (Fig. 8G) brown to red-brown. Endites (Fig. 8G) dark brown and white on inner sides. Labium (Fig. 8G) pale. Sternum (Fig. 8G) heart-shaped, with irregular, median, white stripes. Abdomen (Fig. 8E–G) shield-shaped, dorsum brown, paler anteriorly and darker posteriorly, with several long hairs at the anterior margin, two pairs of muscle depressions medially, and little brown spots anteriorly and laterally; venter dark medially, with a pair of longitudinal white bands laterally. Spinnerets yellow-brown.

**Figure 9.**
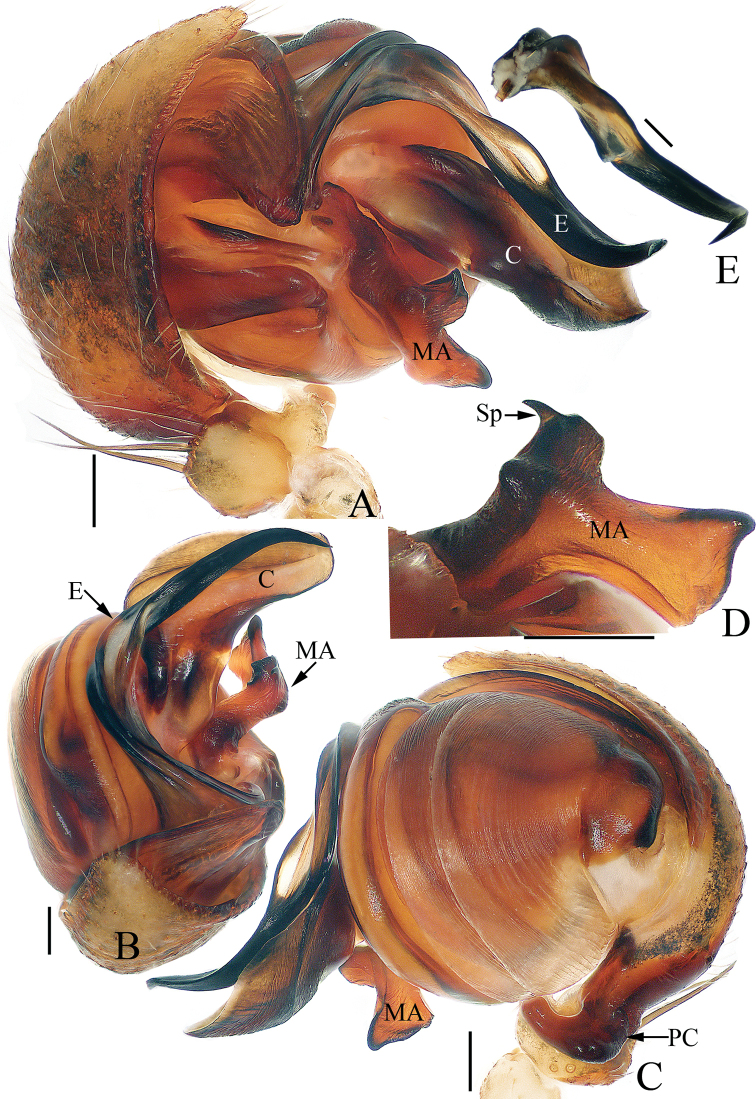
Male palp of *Argiope vietnamensis* Ono, 2010 **A–D**TRU-Araneidae-125 **E** from TRU-Araneidae-131 **A** prolateral **B** apical **C** retrolateral **D** median apophysis, posterior **E** broken embolus found in female’s epigyne, prolateral. Scale bars: 0.1 mm.

Palp (Fig. 9A–D): tibia swollen, with two long dorsal bristles; paracymbium curved 90° medially and column-shaped posteriorly; median apophysis elongated, divided into two branches, and the dorsal one bearing a short spur; conductor ca 3 times longer than wide, touching median apophysis at base; embolus tapered, spiraled, forming ridges anterior-medially, and curved apically to a pointed tip in prolateral view.

###### Distribution.

China (Guizhou, Guangxi); Vietnam.

###### Comments.

A male and a subadult female were collected on the same web from Shiwandashan National Forest Park during the night. The embolus of the male is structurally consistent with the broken embolus found in an *A. vietnamensis* female epigyne. Moreover, other male and females were also collected from Maolan National Nature Reserve. Based on these points of evidence, we propose the males as *A. vietnamensis*.

**Figure 10.**
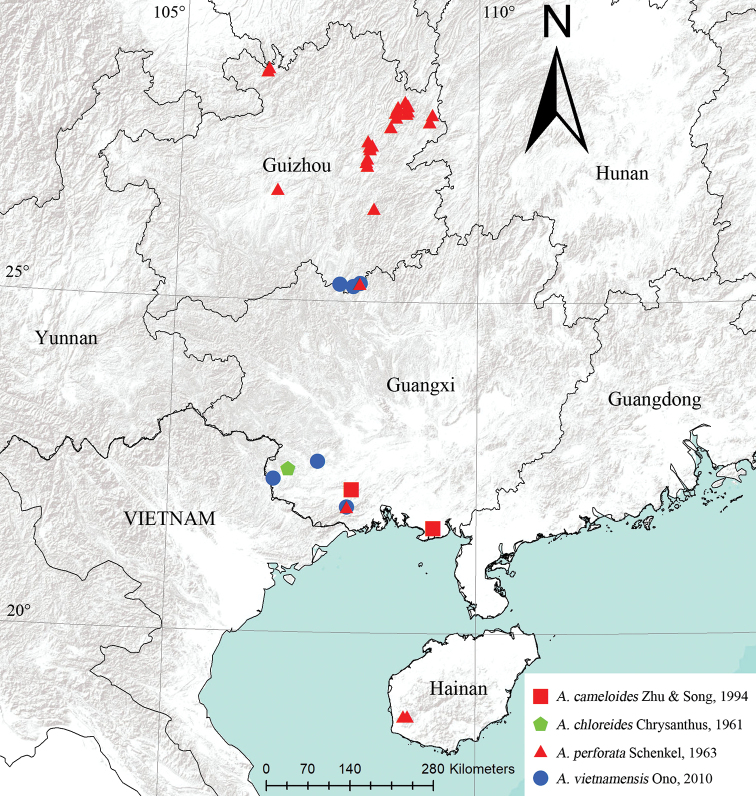
Distributional records of *Argiope* species.

## Supplementary Material

XML Treatment for Argiope cameloides

XML Treatment for Argiope chloreides

XML Treatment for Argiope perforata

XML Treatment for Argiope vietnamensis
